# Complete Genome Sequence of Escherichia coli Podophage Penshu1

**DOI:** 10.1128/MRA.01055-19

**Published:** 2019-09-19

**Authors:** Douglas Pechacek, Myung Hwangbo, Russell Moreland, Mei Liu, Jolene Ramsey

**Affiliations:** a Center for Phage Technology, Texas A&M University, College Station, Texas, USA; b Zachry Department of Civil and Environmental Engineering, Texas A&M University, College Station, Texas, USA; DOE Joint Genome Institute

## Abstract

Escherichia coli 4s is a Gram-negative bacterium found in the equine intestinal ecosystem alongside diverse other coliform bacteria and bacteriophages. This announcement describes the complete genome of the T7-like E. coli 4s podophage Penshu1. From its 39,263-bp genome, 54 protein-encoding genes and a 179-bp terminal repeat were predicted.

## ANNOUNCEMENT

Escherichia coli is a Gram-negative bacterium found living among the intestinal microbiome of all mammals. Due to multiple protective abilities, including extreme acid resistance, E. coli colonizes the intestine as a commensal (1). E. coli 4s was isolated from horse feces and lives among a large diversity of coliform bacteria in the equine gut ecosystem (2). Enteric bacteriophages significantly influence the bacterial composition and exert pathogen suppression (2–4). Here, we describe a newly isolated E. coli podophage called Penshu1.

Penshu1 was isolated from a filtered (filter size, 0.2 μm) wastewater treatment sample from Bryan, TX, using E. coli 4s as the host (2). The phage was propagated using the soft-agar overlay method in Luria broth (BD) under aerobic conditions at 37°C (5). Following isolation, Penshu1 podophage morphology was observed using 2% (wt/vol) uranyl acetate negative staining and transmission electron microscopy performed at the Texas A&M University Microscopy and Imaging Center (6). The phage genomic DNA was extracted as described previously (shotgun library preparation modification of the Promega Wizard DNA clean-up system), and libraries were prepared using an Illumina TruSeq Nano low-throughput kit (7). The DNA was sequenced with an Illumina MiSeq platform as paired-end 250-bp reads using v2 500-cycle chemistry. The resulting 565,076 sequence reads from the index containing the phage genome were quality controlled by FastQC (https://www.bioinformatics.babraham.ac.uk/projects/fastqc/). After trimming using FastX-Toolkit v0.0.14 (http://hannonlab.cshl.edu/fastx_toolkit/), they were assembled into a single contig at 376.4-fold coverage using SPAdes v3.5.0 (8). Contig completion was confirmed by PCR (forward primer, 5′-TGAAGTCTCATGCACTCTTTCC-3′; reverse primer, 5′-CCCTCGTCTATCTTGTGGAATC-3′) and by Sanger sequencing of the resulting product. All of the tools listed here for assembly and annotation were used at default parameters and are available in the Center for Phage Technology Galaxy instance with integrated Web Apollo (https://cpt.tamu.edu/galaxy-pub/) (9, 10). Protein-coding genes were predicted using GLIMMER v3.0 and MetaGeneAnnotator v1.0 (11, 12). No tRNA genes were found after analysis with ARAGORN v.2.36 (13). Protein-coding gene functions were predicted using BLAST v2.2.31 with a 0.001 maximum expectation value, LipoP v1.0, and TMHMM v2.0, and conserved domains were found using InterProScan v5.33-72 (14–17). Rho-independent termination sites were identified using TransTermHP v2.09 (18). Genome-wide DNA sequence similarity comparisons were carried out with progressiveMauve v2.4.0 (19).

Penshu1 has a 39,263-bp genome, with a 93.4% coding density and 50.6% G+C content. Analysis predicted 54 protein-coding genes, with 30 being assigned a putative function. The Penshu1 genome was reopened at T7-like direct terminal repeats of 179 bp predicted by PhageTerm (20). Penshu1 has its highest identity with several unclassified T7-like phages, including 43 similar proteins and 80.3% nucleotide sequence identity with *Escherichia* phage ST31 (GenBank accession number KY962008) and 80.1% nucleotide sequence identity with *Escherichia* phage YZ1 (GenBank accession number MG845865). As for phage T7, Penshu1 has a slippery sequence in the major capsid protein (NCBI accession number QEG09806) that can lead to translation by frameshift of the minor capsid protein (NCBI accession number QEG09807).

### Data availability.

The genome sequence and associated data for phage Penshu1 were deposited under GenBank accession number MK903281, BioProject accession number PRJNA222858, SRA accession number SRR8893626, and BioSample accession number SAMN11414580.
